# Kinetic Analysis of *Yersinia pestis* DNA Adenine Methyltransferase Activity Using a Hemimethylated Molecular Break Light Oligonucleotide

**DOI:** 10.1371/journal.pone.0000801

**Published:** 2007-08-29

**Authors:** Robert J. Wood, Michael D. Maynard-Smith, Victoria L. Robinson, Petra C.F. Oyston, Rick W. Titball, Peter L. Roach

**Affiliations:** 1 School of Chemistry, University of Southampton, Southampton, United Kingdom; 2 Chemical and Biological Sciences, Defence Science and Technology Laboratory, Salisbury, United Kingdom; National Institute on Aging, United States of America

## Abstract

**Background:**

DNA adenine methylation plays an important role in several critical bacterial processes including mismatch repair, the timing of DNA replication and the transcriptional control of gene expression. The dependence of bacterial virulence on DNA adenine methyltransferase (Dam) has led to the proposal that selective Dam inhibitors might function as broad spectrum antibiotics.

**Methodology/Principal Findings:**

Herein we report the expression and purification of *Yersinia pestis* Dam and the development of a continuous fluorescence based assay for DNA adenine methyltransferase activity that is suitable for determining the kinetic parameters of the enzyme and for high throughput screening against potential Dam inhibitors. The assay utilised a hemimethylated break light oligonucleotide substrate containing a GATC methylation site. When this substrate was fully methylated by Dam, it became a substrate for the restriction enzyme DpnI, resulting in separation of fluorophore (fluorescein) and quencher (dabcyl) and therefore an increase in fluorescence. The assays were monitored in real time using a fluorescence microplate reader in 96 well format and were used for the kinetic characterisation of *Yersinia pestis* Dam, its substrates and the known Dam inhibitor, *S*-adenosylhomocysteine. The assay has been validated for high throughput screening, giving a Z-factor of 0.71±0.07 indicating that it is a sensitive assay for the identification of inhibitors.

**Conclusions/Significance:**

The assay is therefore suitable for high throughput screening for inhibitors of DNA adenine methyltransferases and the kinetic characterisation of the inhibition.

## Introduction

N^6^-methylation of adenine in DNA requires *S*-adenosylmethionine (AdoMet) as the methyl group donor and is catalysed by a family of DNA adenine methyltransferases (Dam) found in bacteria and viruses [1], [2]. Dam is a member of this family that selectively modifies the adenine base in the sequence GATC and is responsible for most of the DNA methylation in *Escherichia coli* [3]. Unlike many other DNA adenine methyltransferases, Dam does not form part of a restriction modification system, but it has a pervasive influence in regulating many cellular processes including mismatch repair [4] and the timing of DNA replication [5]. Dam has also been implicated as a global regulator of gene expression: for example the control of the expression of several important genes in *E. coli* [6], [7], the *Yersinia* outer proteins [8] and at least 20 genes in *Salmonella typhimurium* [9].

Manipulating the levels of Dam in bacteria has been shown to reduce bacterial virulence. Dam deficient mutants of *Salmonella typhimurium* [9], *Yersinia pseudotuberculosis* [10] and *Yersinia pestis* [11] show reduction in virulence of 10,000-fold, 1 million-fold and >2300-fold respectively. It has also been observed that inactivation of the *dam* gene is lethal to some bacteria, for example *Y. pseudotuberculosis* YPIII and *Vibrio cholerae* [8].

The degree of attenuation afforded by manipulation of the *dam* gene has led to the proposal that Dam inhibitors could find application as broad spectrum antibiotics [9], [12], [13]. To identify compounds with an inhibitory effect, a reliable assay for Dam activity is required, ideally one readily adapted to high throughput screening. Perhaps the most reliable reported method for measurement of Dam activity involved monitoring the incorporation of a ^3^H labelled methyl group (transferred from ^3^H labelled AdoMet) to an adenine residue within a DNA substrate, typically either calf thymus DNA [14] or a short double stranded oligonucleotide [15]. This method has allowed the kinetic analysis of a number of DNA adenine methyltransferases, including that from *E. coli* [16], [17], bacteriophage T2 [18] and bacteriophage T4 [19], but using this type of discontinuous assay for detailed kinetic studies is labour intensive.

Recently two assays have been reported that couple methylation sensitive restriction enzymes to a fluorescence signal. Both of these assays utilise ‘molecular break light’ oligonucleotides [20]. Based on molecular beacon design [21], they are single stranded, self complementary oligonucleotides which adopt a hairpin loop structure with a fluorophore at the 5′ terminus and a quenching dye at the 3′ terminus. Initially the stem of the oligonucleotide keeps the fluorophore and quencher in close proximity, promoting efficient quenching. Cleavage of the stem leads to separation of the fluorophore and quencher, resulting in an increase in fluorescence which is directly related to the quantity of cleaved DNA. The break light assay has been applied to several examples of DNA modification and cleavage, such as measuring the activity of restriction enzymes [12], nucleases [22], [23] and DNA cleavage by enediynes [20].

By combining the use of methylation sensitive restriction enzymes with a molecular break light oligonucleotide, two alternative assay formats have been developed by Mashhoon *et al*. [12] and Li *et al*. [24]. Mashhoon *et al*. used a break light oligonucleotide in a protection assay. In the first stage, Dam was allowed to methylate the oligonucleotide, and potential Dam inhibitors could be added at this step. In a second step, the oligonucleotide was subjected to cleavage by the restriction endonuclease DpnII (which only restricted the unmethylated DNA). By measuring the resultant fluorescence, the proportion of DNA protected by Dam methylation could be estimated. The assay was suitable for high throughput screening, allowing the relative potency of a library of Dam inhibitors to be compared to a standard. However, the assay is discontinuous and did not permit the rate of methylation to the symmetrical product to be determined.

The principal limitation of this assay is that it is discontinuous, making detailed kinetic analysis rather time consuming. The next logical development was a continuous assay for Dam activity in a format suitable for high throughput analysis. In a recent publication, Li *et al*. [24] described a continuous fluorescence Dam activity assay. This monitored the methylation of an unmethylated break light oligonucleotide in a coupled assay. Two methylation steps are required to make the fully methylated oligonucleotide, which can then be restricted by DpnI, resulting in a proportional increase in fluorescence. Unfortunately, the kinetics of the first methylation to the intermediate hemimethylated oligonucleotide results in a significant lag phase in the observed fluorescence signal, so that the assay is not convenient for determining initial rates of reaction, and Li *et al*. do not report kinetic parameters such as *K*_M_ or *V*_max_ for Dam substrates, or the *K*_i _for the potential Dam inhibitors.

To kinetically characterise Dam and its substrates or inhibitors, it was essential to develop an assay that reported the initial rate of reaction. To do this, we have used a hemimethylated break light oligonucleotide which upon turnover yields the fully methylated product. This is cleaved *in situ* by the restriction enzyme DpnI, resulting in an increase in fluorescence. Having established a direct relationship between the observed fluorescence increase and the methylation activity of Dam with this hemimethylated substrate, we have used the assay to determine the *K*_M_ and *V*_max_ of *Y. pestis* Dam for *S*-adenosylmethionine and the oligonucleotide substrate. To demonstrate the convenience of this assay, we have used it to determine the *K*_i_ of the known Dam inhibitor, *S*-adenosylhomocysteine with *Y. pestis* Dam.

## Results

### Expression and purification of His_6_-tagged *Y. pestis* Dam

Initial attempts at expression of *Y. pestis* Dam in *E. coli* BL21(DE3) resulted in low protein yields and the attenuation of cell growth upon induction with arabinose. As *E. coli* was observed to be very sensitive to the intracellular Dam concentration, Dam was expressed in the *dam-3* and *end-1* strain GM215 [25], which did not show the attenuation of cell growth to the same extent observed in BL21(DE3). Large scale cell growth followed by induction at 37 °C and cell harvest two hours after induction was found to result in the highest yield of active protein. During the optimisation of purification of *Y. pestis* Dam, a rapid thermal inactivation was observed. It was therefore necessary to minimise the time taken to purify the protein, which was shortened to include a single nickel affinity purification step. The highest yield and purity of protein was achieved at pH 9.0. To ensure the purified Dam had the highest possible specific activity, the period of dialysis was kept as short as possible and the majority of the imidazole removed by two short dialysis steps (2×30 min) at 4°C, after which aliquots of protein were flash frozen on dry ice and stored at −80°C. The yield of Dam was consistently 1.5 mg purified protein/l cell culture.

### Continuous break light assay for *Y. pestis* Dam activity

**Figure 1:**
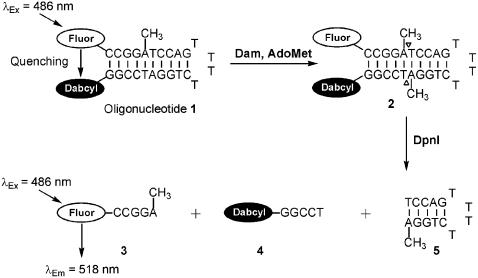
Break light Dam activity assay. The fluorescence of the hemimethylated substrate oligonucleotide 1 is internally quenched by the dabcyl group. It is a substrate for Dam and yields the fully methylated product 2, which is rapidly cleaved by DpnI, thus forming fluorescent oligonucleotide 3.

**Figure 2:**
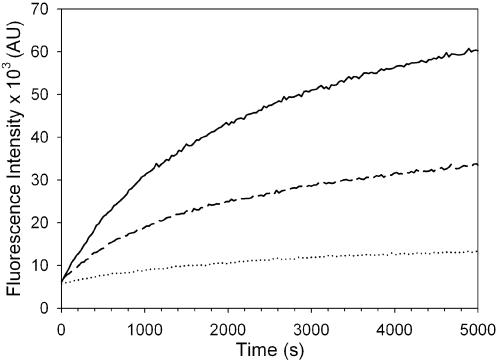
Activity of *Y. pestis* Dam with hemimethylated oligonucleotide substrate. The fluorescence increase was recorded with Dam at the following concentrations: 0 nM (dotted line), 0.31 nM (dashed line) and 0.61nM (solid line).

To allow continuous monitoring of Dam methylation, hemimethylated oligonucleotide **1** (Fig. 1) was used in the coupled enzyme assay. The kinetics of the reaction were greatly simplified by the use of this hemimethylated substrate oligonucleotide, which allowed a single methylation step (Fig. 1, **1**→**2**) to be directly coupled to the DpnI cleavage. The hemimethylated substrate has the added advantage of being the natural substrate for the enzyme during DNA replication [26]. Under the conditions of the assay, DpnI preferentially cleaves doubly methylated oligonucleotide **1** leading to a direct relationship between each single methylation event and fluorescence increase. Example assays are shown in Fig. 2, where the ability of Dam to methylate oligonucleotide **1** was followed by measuring the fluorescence increase in assays containing 0, 0.31 and 0.61 nM Dam and a large excess of DpnI. During the timecourse, the fluorescence intensity increased in the assays containing Dam. Rates of fluorescence increase were highest immediately after initiation, but then slowly decreased. The initial rates of fluorescence increase (up to 180 s) were proportional to the concentration of Dam: for example, at a Dam concentration of 0.31 nM, the rate of fluorescence change was 17.1±2.12 arbitrary units/s, but when the concentration of Dam was doubled to 0.61 nM, the rate of fluorescence also doubled to 34.7±1.82 arbitrary units/s. A background increase in fluorescence was observed in negative control assays (lacking Dam) with an initial rate of fluorescence change of 4.68±1.72 arbitrary units/s. This slow background cleavage is probably due to DpnI cleavage of hemimethylated oligonucleotide **1** [27]. The addition of sodium chloride to the assays has been demonstrated to increase the specificity of DpnI for the doubly-methylated substrate [28], [29]. The sodium chloride concentration was therefore optimised to 20 mM, minimising background cleavage by DpnI in the assay.

### Calibration of oligonucleotide fluorescence

To convert fluorescein emission into a concentration of cleaved oligonucleotide **1**, a calibration curve of DpnI cleaved oligonucleotide **2**, a doubly methylated analogue of oligonucleotide **1**, was plotted. Concentrations of oligonucleotide used were 0–3.5 nM and the reaction endpoint fluorescence was used for the calibration. The variation of fluorescence intensity with concentration of oligonucleotide was fitted to a linear function (*R* ^2^ = 0.998), and the measured gradient of oligonucleotide **2** fluorescence increase was 9.3×10^3^±0.3×10^3^ arbitrary units/nM.

### Effect of substrates on Dam activity

**Figure 3:**
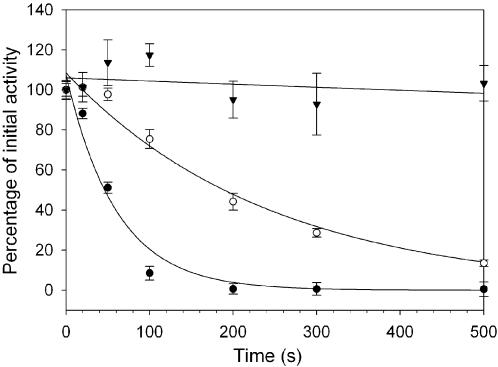
Inactivation of *Y. pestis* Dam. Dam was incubated in a PCR machine at 30 °C with the hemimethylated substrate oligonucleotide (triangles), with AdoMet (open circles) or with no additions (filled circles). Aliquots were withdrawn at time intervals and the activity measured. Activity is expressed as a percentage of the initial activity.

*Y. pestis* Dam was observed to be sensitive towards inactivation, and it was of interest to determine the kinetics of this process and to investigate the ability of substrates to stabilise the enzyme. To better understand this inactivation process, Dam was incubated for 0–500 s at 30°C, without substrates or in the presence of either 30 nM oligonucleotide **1** or 120 µM AdoMet. Aliquots of Dam were withdrawn throughout the time course and then assayed for activity. The first order rate constants (*k*_inact_) for enzyme inactivation were obtained by fitting the results to an exponential function (Fig. 3) and half lives calculated from t_½_ = 0.69/*k*_inact_.

When incubated alone, Dam was very unstable, with a half life of 42.5±7.5 seconds. Both substrates had a protective effect on the methyltransferase activity, increasing the half life of the enzyme by 3–50 fold. Incubation with oligonucleotide **1** had the greatest protective effect increasing the enzyme half life to >2300 seconds. At least one other example of methyltransferase instability has been reported, for the *Caulobacter crescentus* cell cycle regulated adenine N-6 methyltransferase (Ccrm), for which a *k*_inact _of 2.3×10^−3^ s^-1^ at 30°C was observed in the absence of substrates [30]. CcrM was also stabilized by the addition of a saturating concentration of DNA.

### Kinetic analysis of *Y. pestis* Dam using a break light assay

**Figure 4:**
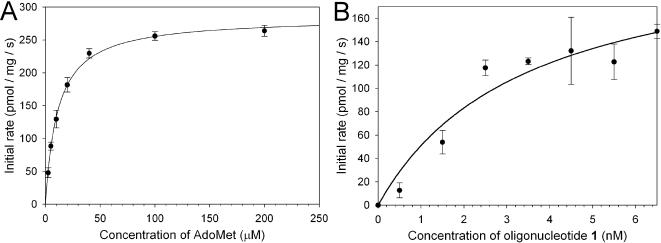
Dependence of Dam activity on substrate concentration. (A) AdoMet and (B) oligonucleotide 1.

Our break light assay was used to determine *K*_M_^AdoMet^ and *K*_M_^DNA^ for oligonucleotide **1**. The dependence of Dam activity on AdoMet concentration was fitted to a hyperbola (Fig. 4A). The measured *K*_M_^AdoMet^ was 11.3±0.63 µM, and is two times higher than that reported for *E. coli* Dam [17] (Table 1).

**Table 1:** A comparison of *Y. pestis* and *E. coli* Dam kinetic parameters.

Source of Dam	*Y. pestis*	*E. coli*	*E. coli*
Substrate	10 bp Hairpin	double-stranded 20-mer [17]	16 bp Hairpin [17]
*K*_M_ AdoMet (µM)	11.3±0.63	5.60±1.10	6.40±1.20
*K*_M_ DNA (nM)	3.43±1.68	17.4±3.0	28.6±0.16
*K*_i_ SAH (µM)	6.93±2.01	41.6±10.4	Not Detectable
*k*_cat_ (min^−1^)	0.55±0.01	0.93±0.06	1.19±0.33

Kinetic data for *Y. pestis* Dam was obtained from a SigmaPlot fitted hyperbola of the form *v* = *V*_max_ [S]/*K*_M_+[S].

For the DNA substrate, data were also fitted to a hyperbola (Fig. 4B) to give *K*_M_^DNA^ = 3.43±1.68 nM. The *Y. pestis* enzyme shows a considerably lower *K*_M_^DNA^ than that reported for the *E. coli* protein [17]. The observed *k*_cat_ for *Y. pestis* Dam is 0.55±0.01 min^−1^, a similar rate to that reported for both the *E. coli* and T4 DNA adenine methyltransferases [31]. The error in *K*_M_^AdoMet ^and *k*_cat_ were comparable to those obtained using the tritium labelled filter binding assay. The observed instability of *Y. pestis* Dam in the absence of high concentrations of stabilising DNA makes the determination of the *K*_M_^DNA^ inherently more difficult and this is reflected in the relatively large observed error.

### Measurement of *K*_i_ of *S*-adenosylhomocysteine for *Y. pestis* Dam

**Figure 5:**
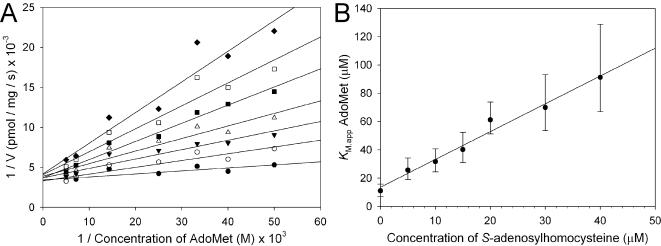
Inhibition of Dam by *S*-adenosylhomocysteine. (A) A double reciprocal plot of methylation rate against AdoMet concentration. *S*-adenosylhomocysteine concentrations were; 0 (•), 5 (○), 10 (▾), 15 (Δ), 20 (▪), 30 (□) and 40 (♦) µM. (B) Apparent *K*_M_ plotted against the concentration of *S*-adenosylhomocysteine.

The effect of a known Dam inhibitor, *S*-adenosylhomocysteine on *Y. pestis* Dam was investigated using the break light assay. From the data shown in Fig. 5, the *K*_i_ was calculated to be 6.93±2.01 µM and is lower than the reported value for the *E. coli* enzyme (*K*_i_ = 41.6±10.4 µM). These experiments demonstrate the application of the assay to the detailed kinetic analysis of a single inhibitor.

### High throughput screening assay validation

**Figure 6:**
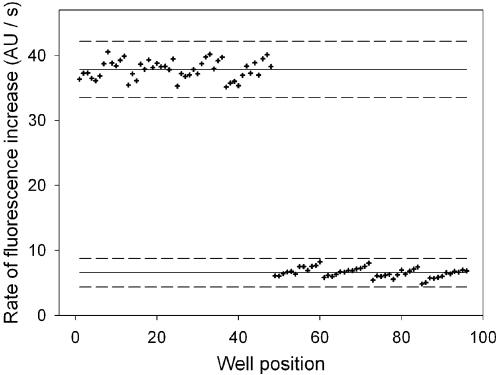
High throughput assay validation. Rate of fluorescence increase was monitored over 96 wells (wells 1–48 contained positive control assays, wells 49–96 contained negative control assays with no Dam). The solid trend lines indicate the mean rate of fluorescence increase for the positive/negative control datasets; the broken lines indicate the ±3 standard deviations from the mean, which is the 99.7% confidence limit.

The use of the hemimethylated oligonucleotide substrate for high throughput screening was validated using the method of Zhang [32]. Data were collected from three 96 well plates containing positive (normal assay conditions, no inhibitor) and negative controls (assays containing no Dam) to assess the reproducibility and distribution the rate of fluorescence increase at the two activity extremes. From the distribution of the rates of fluorescence increase (Fig. 6), the 99.7% confidence limit (3 standard deviations from the mean) for control assays show that the assay has a high separation band, indicating that it is a highly sensitive assay for inhibitor identification. The data gives an average screening window coefficient (Z-factor) of 0.71±0.07 over three 96 well plates, implying that it is an excellent assay for hit detection.

## Discussion

Our initial investigations into the use of a DpnI cleavage assay to monitor Dam methylation began with a non-methylated break light oligonucleotide using a similar substrate to that of Li *et al.* [24]. We found that the methylation kinetics of this substrate were too complicated to be simply resolved to allow a detailed kinetic analysis of the Dam methylation reaction. To kinetically characterise Dam and its substrates or inhibitors, it was essential to develop an assay that reported the initial rate of reaction. To do this, we have used a hemimethylated break light oligonucleotide which upon turnover yields the fully methylated product. This is cleaved *in situ* by the restriction enzyme DpnI, resulting in an increase in fluorescence. Having established a direct relationship between the observed fluorescence increase and the methylation activity of Dam with this hemimethylated substrate, we have used the assay to determine the *K*_M_ and *V*_max_ of *Y. pestis* Dam for AdoMet and the oligonucleotide substrate. To demonstrate the convenience of this assay, we have used it to determine the *K*_i_ of the known Dam inhibitor, *S*-adenosylhomocysteine with *Y. pestis* Dam.

This new approach has distinct practical advantages: the assay is simple to use, highly reproducible and gives real time continuous data which correlates directly to each methylation event giving the potential to allow more accurate determination of initial rates over shorter time scales. It permits the rapid determination of kinetic parameters and the *K*_i_ for potential inhibitors, providing a safer alternative to the radioactive filter binding assay.

The assay has been validated for high throughput screening, giving a Z-factor of 0.71±0.07, indicating that it is a highly sensitive and reproducible assay for hit detection and is sufficiently flexible to permit its application to screening large compound libraries for Dam inhibitors.

## Materials and Methods

### Materials

Expression vector pBAD/HisA and *E. coli* TOP10 competent cells were obtained from Invitrogen (Groningen, NL). DpnI was obtained from New England Biolabs (Herts., UK). *Y. pestis* was manipulated using suitable biological safety precautions and containment facilities. *Y. pestis* genomic DNA was isolated from *Y. pestis* strain GB using a PureGene DNA isolation kit from Gentra Systems (Minneapolis, USA). Other DNA was isolated from cultures or agarose gels using commercially available kits following the manufacturers instructions. *E. coli* GM215 was obtained from the Yale *E. coli* genetic stock centre (CGSC# 6645). PCR primers were purchased from Sigma-Genosys (Haverhill, UK). For fluorescence measurements, oligonucleotides **1** and **2** were purchased from ATDBio (Southampton, UK), flat bottomed black 96 well polypropylene and half area flat bottomed 96 well polystyrene microplates were purchased from Greiner Bio-One Ltd (Stonehouse, UK) and measurements were taken using a Tecan Safire 2 microplate reader (Reading, UK). Assays for high throughput screening were prepared using a Beckman Coulter Biomek 3000 liquid handling system equipped with a 200 µl single channel and 20 µl 8 channel pipette head. Mineral oil (sterile filtered, mouse embryo tested, light oil) and *S*-adenosylmethionine chloride were obtained from Sigma Aldrich (Poole, UK). Bovine serum albumin (BSA) was purchased from Advanced Protein Products Ltd (Brierley Hill, UK).

### Construction of pRJW4213/07 for the expression of *Y. pestis* Dam

DNA manipulations were carried out using standard protocols [33]. The *dam* gene was amplified by PCR from *Y. pestis* GB genomic DNA using *Pfu* Turbo polymerase and the following oligonucleotide primers: *pfdam* 5′ GGCGGC*CCATGG*GC**CACCACCACCACCACCAC**AAGAAAAACCGCGCTTTTTTAAAATGG and *prdam* 5′ GCCGCC*AAGCTT*TCAGCTATAGAGCGCCAAAAG. The *dam* gene was amplified adding an NcoI site (italics) and DNA encoding a His_6_tag (bold) to the 5′ end of the amplified product, and a HindIII site (italics) at the 3′ end. The PCR amplification product was purified and the NcoI-HindIII fragment containing the modified *dam* gene inserted by ligation between unique NcoI and HindIII sites of pBAD/HisA to yield plasmid pRJW4213/07.

### Expression and purification of *Y. pestis* Dam

All purification steps were carried out at 4°C and samples centrifuged in a Beckman JA-14 rotor unless otherwise stated. All cell culture media contained 100 µg/ml ampicillin. Cell pastes and purified proteins were stored at −80°C. Protein purity was judged by SDS-PAGE with Coomassie staining.

2YT medium (100 ml) was inoculated from stored strains (pRJW4213/07 in *E. coli* strain GM215) and grown overnight in a shaking incubator at 37°C and 180 rpm. This overnight culture was used as 1 % innocula into 4×1250 ml fresh medium and grown until the OD_600_ reached 0.6. The cultures were induced by addition of 10 ml/l of a filter sterilised 20 % w/v arabinose solution and growth continued at 37°C for two hours. Cells were harvested by centrifugation at 8000 rpm, 4°C, for 12 minutes and the cell paste, typically 20 g was stored at −80°C until required.

Dam was purified from 10 g of cell paste resuspended in 30 ml of buffer A [50 mM Tris/HCl (pH 9.0), 50 mM imidazole, 300 mM NaCl, 0.05 % v/v triton X-100, 10 % w/v glycerol, 10 mM 2-mercaptoethanol] and 0.3 ml of a 10 mg/ml lysozyme solution added. The suspension was stirred for 20 minutes at 4°C and then sonicated 25 times for 5 second bursts. The lysate was cleared by centrifugation at 12,000 rpm and 4°C for 30 min. The supernatant was applied to a nickel charged chelating sepharose FF column (5 ml bed volume) previously equilibrated in buffer A. The column was washed with 200 ml of buffer A and the proteins eluted with a 30 ml gradient to 100 % buffer B (buffer A plus 500 mM imidazole). The purest fractions (8 ml) were pooled and dialysed twice for 30 min at 4°C against 500 ml buffer C [50 mM Tris/HCl (pH 7.5), 200 mM NaCl, 0.2 mM EDTA, 20 % w/v glycerol, 2 mM dithiothreitol]. The purification yielded 1.5 mg Dam from 10 g cell paste and aliquots of protein solution (100 µl) were immediately frozen at −80°C.

### Dam activity assay

Fluorescence changes were recorded in a Tecan Safire 2 microplate reader using 10 readings per well (each measurement), 0.5 s between each movement and reading, 1 second of shaking between data collections with 7 seconds of settle time. The following instrument settings were used: the excitation wavelength was 486 nm, the emission wavelength was 518 nm, the bandwidth was 5 nm, the gain was 200, the Z-position was 9300 µm and the integration time was 40 µs. Break light oligonucleotide sequences used in the assay were; oligonucleotide **1**: 5′ C(F)CG**GA^m^TC**CAGTTTTCTG**GATC**CGG(D) 3′; oligonucleotide **2**: 5′ C(F)CG**GA^m^TC**CAGTTTTCTG**GA^m^TC**CGG(D) 3′, where Dam recognition sequences are shown in bold, (F) represents fluorescein and (D) represents a dabcyl quencher.

The activity of Dam was measured in triplicate in Greiner flat bottomed black 96 well polypropylene microplates, with a total assay volume of 200 µl, maintained at 37°C. Three buffers were required: buffer D [22.4 mM Tris-acetate (pH 7.9), 23.5 mM sodium chloride, 56 mM potassium acetate, 11.2 mM magnesium acetate, 1.12 mM dithiothreitol, 0.12 mg/ml BSA] which could be modified by the addition of between 0 and 235 µM AdoMet and between 0 and 35 nM oligonucleotide **1**; buffer E [20 mM Tris-acetate (pH 7.9), 50 mM potassium acetate, 10 mM magnesium acetate and 1 mM dithiothreitol]; buffer F [48 mM Tris/HCl (pH 7.4), 9.6 mM EDTA, 4.8 mM 2-mercaptoethanol, 0.4 mg/ml BSA and 30 nM oligonucleotide **1**]. Dam solution was prepared by defrosting an aliquot of Dam stock (as purified, 0.17 mg/ml) on ice for 10 minutes and diluting it 510 fold into buffer F. DpnI solution was prepared by diluting the stock solution to 1 U/µl in buffer E.

170 µl of buffer D was added to each well of the plate and overlaid with 3 drops of mineral oil. The plate was then equilibrated at 37°C for 15 minutes and the reaction initiated by the addition of 10 µl of DpnI solution and 20 µl of Dam solution to each well. Fluorescein emission was then monitored over time. The rate of reaction was calculated by taking the initial rate of fluorescence change over 180 seconds unless otherwise stated. Background changes in fluorescence were accounted for by subtracting a negative control (lacking Dam) when appropriate. The rate of change in fluorescence was converted to a rate of reaction using a fluorescence calibration curve. Data were fitted with the program SigmaPlot.

### Oligonucleotide 1 fluorescence calibration curve

Assays contained the fully methylated oligonucleotide **2** in place of **1** at concentrations 0, 0.5, 1, 2, 3, 3.5 nM, 25 µM AdoMet and no Dam. The endpoint fluorescence was measured.

### Kinetic analysis of *Y. pestis* Dam

The dependence of enzyme activity on AdoMet concentration was measured in a series of assays containing 33 nM oligonucleotide **1**, 1.0 nM Dam and 2.5, 5, 10, 20, 40, 100, 200 µM AdoMet. The dependence of reaction rate on oligonucleotide **1** concentration was measured in a series of assays containing 0.31 nM Dam, 120 µM AdoMet and 0.5, 1.5, 2.5, 3.5, 4.5, 5.5, 6.5 nM oligonucleotide **1**.

### Inactivation of *Y. pestis* Dam

The inactivation was monitored at 30°C under three conditions: in the absence of substrates, with 30 nM oligonucleotide **1** or with 120 µM AdoMet. The Dam solution was prepared by diluting Dam (as purified) 213 fold in buffer F lacking DNA. To 200 µl of Dam solution was added 100 µl of DpnI solution and the resultant mixture aliquotted into PCR tubes (35 µl in each). The PCR tubes were maintained at 30°C in a PCR machine and aliquots withdrawn at the required time points. Aliquots were then rapidly cooled in an ice bath and the activities assayed at the end of the timecourse.

### Inhibition of Dam by *S*-adenosylhomocysteine

Reactions contained 1 nM Dam and 33 nM oligonucleotide **1**, with varying AdoMet and *S*-adenosylhomocysteine concentrations: *S*-adenosylhomocysteine; 0, 5, 10, 15, 20, 30, 40 µM, AdoMet; 20, 25, 30, 40, 70, 140, 200 µM. *K*_i_^SAH^ was estimated as follows: a double reciprocal plot (Fig. 5A) of 1/rate of reaction against 1/concentration of AdoMet has a slope of *K*_M.app_/*V*_max_, yielding a series of values for the apparent *K*_M_ (*K*_M.app_) at different inhibitor concentrations. A plot of *K*_M.app _against concentration of *S*-adenosylhomocysteine (Fig. 5B) has intercepts of *K*_M_ on the *K*_M.app _axis and –*K*_i_ on the concentration of *S*-adenosylhomocysteine axis.

### High throughput screening validation conditions

The activity of Dam was measured in half area Greiner flat bottomed black 96 well polystyrene microplates, with a total assay volume of 100 µl, maintained at 30°C. Four buffers were required: buffer G [buffer D containing 5.9 µM AdoMet and 35 nM oligonucleotide **1** (final concentrations of 5 µM and 33 nM respectively in the assay)]; buffer H [buffer E containing 1 U/µl DpnI (final concentration 5 U per assay)]; buffer I [buffer F containing 20 nM Dam (for a final concentration of 2 nM)]. Per 96 well plate, 1160 µl buffer I was mixed with 580 µl buffer H to make buffer J.

Using a Biomek 3000 liquid handling system, 85 µl of buffer G was added to each well. The plate was then equilibrated at 30°C for 20 minutes and the reaction initiated by the addition of 15 µl of buffer J per well. The plate was then immediately transferred to a Tecan Saffire 2 microplate reader and fluorescein emission monitored over time. The following instrument settings were used: 10 readings per well (each measurement), 0 s between each movement and reading and no shaking between data collections. The excitation wavelength was 486 nm with a bandwidth of 9 nm, the emission wavelength was 518 nm with a bandwidth of 20 nm, the gain was 130, the Z-position was 9300 µm and the integration time was 40 µs. The rate of reaction was calculated by taking the initial rate of fluorescence change over the first 675 seconds. The rate of fluorescence increase was estimated by fitting data to a linear trend line with the program Excel.
